# The Role of a Composite Fitness Score in the Association Between Low-Density Cholesterol and All-Cause Mortality in Older Adults: An Individual Patient Data Meta-Analysis

**DOI:** 10.1093/gerona/glad148

**Published:** 2023-06-14

**Authors:** Milly A van der Ploeg, Rosalinde K E Poortvliet, Jonathan M K Bogaerts, Veerle M G T H van der Klei, Ngaire Kerse, Anna Rolleston, Ruth Teh, Louise Robinson, Carol Jagger, Yasumichi Arai, Ryo Shikimoto, Yukiko Abe, Jeanet W Blom, Yvonne M Drewes, Jacobijn Gussekloo

**Affiliations:** Department of Public Health and Primary Care, Leiden University Medical Center, Leiden, The Netherlands; Department of Public Health and Primary Care, Leiden University Medical Center, Leiden, The Netherlands; Department of Public Health and Primary Care, Leiden University Medical Center, Leiden, The Netherlands; Department of Internal Medicine, Section of Gerontology and Geriatrics, Leiden University Medical Center, Leiden, The Netherlands; School of Population Health, University of Auckland, Auckland, New Zealand; The Centre for Health, Tauranga, New Zealand; School of Population Health, University of Auckland, Auckland, New Zealand; Population Health Sciences Institute, Newcastle University, Newcastle upon Tyne, UK; Population Health Sciences Institute, Newcastle University, Newcastle upon Tyne, UK; Center for Supercentenarian Medical Research, Keio University School of Medicine, Tokyo, Japan; Center for Supercentenarian Medical Research, Keio University School of Medicine, Tokyo, Japan; Center for Supercentenarian Medical Research, Keio University School of Medicine, Tokyo, Japan; Department of Public Health and Primary Care, Leiden University Medical Center, Leiden, The Netherlands; Department of Public Health and Primary Care, Leiden University Medical Center, Leiden, The Netherlands; Department of Internal Medicine, Section of Gerontology and Geriatrics, Leiden University Medical Center, Leiden, The Netherlands; Department of Public Health and Primary Care, Leiden University Medical Center, Leiden, The Netherlands; Department of Internal Medicine, Section of Gerontology and Geriatrics, Leiden University Medical Center, Leiden, The Netherlands

**Keywords:** Activities of daily living, Cognition, Frailty, Grip strength, Oldest old

## Abstract

**Background:**

In the general population, an increase in low-density lipoprotein cholesterol (LDL-C) predicts higher cardiovascular disease risk, and lowering LDL-C can prevent cardiovascular disease and reduces mortality risk. Interestingly, in cohort studies that include very old populations, no or inverse associations between LDL-C and mortality have been observed. This study aims to investigate whether the association between LDL-C and mortality in the very old is modified by a composite fitness score.

**Methods:**

A 2-stage meta-analysis of individual participant data from the 5 observational cohort studies. The composite fitness score was operationalized by performance on a combination of 4 markers: functional ability, cognitive function, grip strength, and morbidity. We pooled hazard ratios (HR) from Cox proportional-hazards models for 5-year mortality risk for a 1 mmol/L increase in LDL-C. Models were stratified by high/low composite fitness score.

**Results:**

Composite fitness scores were calculated for 2 317 participants (median 85 years, 60% females participants), of which 994 (42.9%) had a high composite fitness score, and 694 (30.0%) had a low-composite fitness score. There was an inverse association between LDL-C and 5-year mortality risk (HR 0.87 [95% CI: 0.80–0.94]; *p* < .01), most pronounced in participants with a low-composite fitness score (HR 0.85 [95% CI: 0.75–0.96]; *p* = .01), compared to those with a high composite fitness score (HR = 0.98 [95% CI: 0.83–1.15]; *p* = .78), the test for subgroups differences was not significant.

**Conclusions:**

In this very old population, there was an inverse association between LDL-C and all-cause mortality, which was most pronounced in participants with a low-composite fitness scores.

Atherosclerotic cardiovascular diseases (ACVD) are highly prevalent in advanced age and an important cause of morbidity and mortality (1). Lowering low-density cholesterol (LDL-C) level is an important strategy in cardiovascular disease prevention. Results from clinical trials have shown that lowering LDL-C with medication effectively reduces the number of cardiovascular events and decreases mortality risk (2–4). Also, an increase in LDL-C is linearly associated with cardiovascular disease risk (5).

However, observational research on the relation between LDL-C and all-cause mortality has offered conflicting results. Most observational studies performed in older populations report no, or inverse associations (6–9). These associations are counter-intuitive, as we would expect high LDL-C to predict greater mortality risk. We hypothesize that a person’s level of fitness modifies this association between LDL-C and mortality. A similar phenomenon was observed in a study (10) that examined the association between systolic blood pressure and mortality, where the association with blood pressure was dependent on a performance on a measure of physical fitness. Also, markers of fitness generally predict mortality in (frail) older adults better than traditional cardiovascular risk markers (8,11,12).

Therefore, this study aims to investigate whether the association between LDL-C and mortality in the older is modified by fitness, operationalized with a composite fitness score. In this meta-analysis, we take the opportunity of combining individual participant data from several studies of people of advanced age, across nations, and cultural groups to examine the stability of associations around the world.

## Method

### Design and Study Population

This 2-stage individual participants data (IPD) meta-analysis includes data from 5 cohorts of older adults from the 4 studies of the Towards Understanding Longitudinal International older People Studies (TULIPS) Consortium: (1) the Leiden 85-plus Study; (2) Te Puāwaitanga O Ngā Tapuwae Kia Ora Tonu: Life and Living in Advanced Age, a cohort study in New Zealand (LiLACS-NZ), providing a (2a) Māori (Indigenous people of NZ), and (2b) non-Māori cohort; (3) the Newcastle 85+ Study; and (4) the Tokyo Oldest Old Survey on Total Health (TOOTH).

The selection criteria for this analysis were the availability of either fasting or nonfasting LDL-C measurements at baseline. We excluded participants who had had a baseline triglyceride measurement of ≥4.5 mmol/L (or ≥400 mg/dL), as high triglyceride levels may distort LDL-C levels. To avoid effect overestimation due to the association between terminal illness and progressively declining LDL-C, we also excluded participants who died within the first 90 days of follow-up. For all studies, ethical approval was obtained from their respective authorities. Informed consent was obtained from all participants for data collection and publication of the results. Details on measures and data collection have been previously reported (13–17).

#### The Leiden 85-plus Study

In this study, all participants were recruited from the city of Leiden, The Netherlands. Of the 705 residents who reached the age of 85 between September 1997 and September 1999, 599 were enrolled in the study of which 556 had LDL-C levels measured at baseline (17).

#### The LilACS-NZ study

This study consists of 2 cohorts, 1 with Māori and 1 with non-Māori participants. Participants aged 80–90 years were recruited for the Māori cohort due to disparity in longevity and to enable equal explanatory power for analyses, and participants aged 85 years for the non-Māori cohort were recruited from the area within the boundaries of the Bay of Plenty and Lakes District Health Board (excluding Taupo area), in 2010. Of the total age-eligible (*n* = 1 636), 421 Māori and 516 non-Māori participants were enrolled (15,16). Baseline LDL-C levels were available for 201 Māori participants and for 348 non-Māori participants.

#### The Newcastle 85+ Study

Participants for the Newcastle 85+ Study were recruited in 2006. All inhabitants who were born in 1921 and registered with a participating general practitioner in Newcastle upon Tyne or North Tyneside primary care trusts, the United Kingdom, were approached for participation (*n* = 1 470). There were 849 participants enrolled in the study with complete health assessment and record review (14), of which 775 participants had LDL-C levels measured.

#### The TOOTH study

Baseline data were collected between March 2008 and November 2009. A random sample of inhabitants of the Shinjuku, Minato, and Shibuya wards in city of Tokyo, aged 85 years or older, was drawn from the basic city registry. Of 2 875 eligible individuals, a total of 1 152 people were recruited, of which 542 completed both the in-home interview and the medical/dental examination (13). Baseline LDL-C levels were available for 538 of the 542 participants.

### Participants

A total of 2 927 participants were enrolled in the 5 cohorts, of which 2 418 participants had LDL measurements. Six participants were excluded because of baseline triglyceride measurement of >4.5 mmol/L, and another 22 participants were excluded because they died in the first 90 days of follow-up. Five participants were excluded because of a loss to follow-up. This left a total of 2 385 participants that were included in the analyses.

### Data Collected at Baseline

#### Sociodemographic characteristics

In all cohorts, baseline data per participant on age and sex were collected.

#### Cholesterol-lowering medication

In all cohorts, the use of one or more lipid-lowering drugs (drugs indexed as C10 by the Anatomical Therapeutic Chemical Classification System of the World Health Organization (18)), at baseline was recoded into yes/no.

#### Cholesterol

In the LilACS-NZ study and Newcastle 85+ Study total cholesterol, high-density lipoproteins (HDL-C), LDL-C, and triglycerides were measured in venous blood samples after an overnight fast (14,16). In the Leiden 85-plus Study total cholesterol, HDL-C, and triglycerides were measured in a nonfasting venous blood sample and the Friedewald formula was used to calculate LDL-C (19,20). In the TOOTH study, total cholesterol, HDL-C, LDL-C, and triglycerides were measured in venous nonfasting blood samples.

#### Individual markers of fitness

There were 4 individual markers of fitness that were available in all cohorts, and that could be included in this study to define subgroups. Details on the variables used in the analyses are shown in Supplementary Table 1.

1) Functional ability: In the Leiden 85-plus Study, functional ability was measured with the Groningen Activity Restriction Scale (GARS, range 18–72) (21). In the LilACS-NZ cohorts, the Nottingham Extended Activities of Daily Living (NEADL) index was used (range 0–15) (22). In the Newcastle 85+ Study, a disability sum score was used (range 0–17). In the TOOTH study, the Lawton Instrumental Activities of Daily Living scale (Lawton iADL) was used (range 0–5). For analyses, we reversed the polarity of the GARS and the disability sum score that was used in the Newcastle 85+ Study, so that on all scales higher scores indicate better functional ability. A score in the low tertile (T1) of the cohort was considered a marker of low levels of fitness, and a score in the high tertile (T3) of the cohort was considered a marker of high level of fitness.
2) Cognitive function: In all cohorts, cognitive function was assessed with the Mini-Mental State Examination (MMSE) questionnaire (maximum score of 30). Higher MMSE scores indicate better cognitive functioning (23). A score in the low tertile (T1) of the cohort was considered a marker of low levels of fitness, and a score in the high tertile (T3) of the cohort was considered a marker of high levels of fitness.
3) Grip strength: Handgrip strength was measured in kilograms by the use of handheld dynamometers. Measurements of grip strength in the low sex-specific tertile (T1) cohort or inability to perform the test were considered a marker of low levels of fitness and in the high sex-specific tertile (T3) of cohort a marker of the high levels of fitness.
4) Morbidity: Three chronic diseases with relevance for survival risk and LDL-C, that were assessed comparable in the 4 studies, were included: diabetes mellitus (diabetes), malignancy (excluding non-melanoma skin malignancies), and history of ACVD (angina pectoris, myocardial infarction, intermittent claudication, transient ischemic attack, stroke, or related surgeries). Information on these diseases was obtained from the interviews and/or general practitioner records, and from electrocardiograms. We considered having ≥2 of these three morbidities (high morbidity), a marker of a low levels of fitness, and the absence of all 3 morbidities (low morbidity), a marker of high levels of fitness.

Cutoff values for the tertiles of functional ability, cognitive function, and grip strength, were chosen in a way that the number of participants that fell in the groups was closest to 33% of the cohort size.

#### Composite fitness score

We combined the 4 individual markers of fitness (functional ability, cognitive function, grip strength, and morbidity) into the composite fitness score, and pragmatically defined a low-composite fitness score as having ≥2 of the 4 markers of low level of fitness, and a high composite fitness score as having ≥2 of markers as high level of fitness. Participants that did not meet these criteria, or who had 2 individual markers as high and 2 individual markers as low level of fitness, were classified as rest group. These criteria best served the prespecified aim to compare participants with 33% highest and 33% lowest level of fitness in each cohort.

#### All-cause mortality

For the Leiden 85-plus Study, LilACS-NZ study and the Newcastle 85+ Study, mortality data were obtained from national registries (14,15,17). For the TOOTH study, death status was ascertained by annual telephone calls (13). Survival time over 5 years was calculated as the time between the baseline visit and date of death, or censored at 1 826 days (5 years).

### Statistical Analyses

In the first stage, the analyses were performed at the cohort level. In the second stage, we pooled the cohort-level results.

#### Cohort-level analyses

We present continuous variables as mean with standard deviation (*SD*) or as median with interquartile range (IQR) depending on the distribution of the data. Categorical variables are presented as frequencies with the percentage of the valid total. To test for group differences between categories of participants, we used independent *t* tests or Mann–Whitney *U*-tests for continuous variables, and the chi-square tests for categorical variables. We carried out a 5-year survival analysis using Cox proportional hazards regression models. We calculated hazard ratios (HRs) with 95% confidence intervals (CIs) per mmol/L change in LDL-C level, adjusted for age, sex, and use of lipid-lowering medication (initial model).

Using the initial model, we first stratified by the individual fitness markers (T3/T1) and finally by the composite fitness score (high/low). We tested for subgroup differences with the Chi-square test between the strata.

#### Pooled analysis

With the results of the cohort-level analyses, we performed pooled analyses using random-effects models with inverse-variance weighting. Inconsistency between cohorts due to heterogeneity was quantified using the I^2^-statistic, I^2^ below 40% was defined as consistency between estimates (24). The cohort-level analyses were performed using IBM Statistical Package for the Social Sciences (SPSS) Statistics version 25.0 (IBM, Armond, NY). The pooled analyses were performed using Review Manager 5.4.1 (The Cochrane Collaboration, Copenhagen, Denmark, 2020). A 2-sided *p* value of ≤.05 was considered statistically significant.

#### Sensitivity analyses

We conducted 3 sensitivity analyses: (1) we assessed whether the association between LDL-C and mortality was similar for participants with and without ACVD, by stratification, (2) to have a closer look at the role of lipid-lower medication use in the association between LD-C and mortality in the subgroups of the composite fitness score, we double stratified by composite fitness score (high/low) and by lipid-lowering medication use (yes/no), and adjusted for age and sex, and (3) we repeated the initial analyzes with total cholesterol instead of LDL-C. These analyses were also performed in a 2-stage approach.

## Results

In Table 1, the baseline characteristics of the 2 385 included participants of the 5 TULIP Consortium cohorts are described. The median age was 85 years (IQR 85.1–85.9) and 59.2% were female participants. Use of lipid-lowering medication ranged from 1.1% in the Leiden cohort, to 47.9 % in the LilACS-NZ non-Māori cohort. Of the total population, 879 participants (37.5%) were free from ACVD, diabetes, and malignancy (ranging from 22.2% of the LiLACS-NZ Māori cohort to 49.8% of the TOOTH cohort), and 367 (15.5%) had ≥2 of these morbidities (ranging 7.9% of the TOOTH, to 30.8% of the LiLACS-NZ Māori cohort). The mean total cholesterol was 5.2 mmol/L (*SD* 1.1), the mean HDL was 1.5 mmol/L (*SD* 0.4), and the mean LDL-C was 3.0 mmol/L (*SD* 1.0). The overall median MMSE score was 27 (IQR 25–29), the median grip strength for females was 17 kg (IQR 14–20), and 28 kg (IQR 23–33) for male participants.

**Table 1.** Characteristics of 2 385 Participants With LDL-C Level Measurement at Baseline of the 5 TULIP Consortium Cohorts

Study Population	Leiden 85-plus	LilACS-NZ		Newcastle 85+	TOOTH	Total
		a. Māori	b. non-Māori			
*N*	(*n* = 547)	(*n* = 199)	(*n* = 347)	(*n* = 767)	(*n* = 525)	(*n* = 2 385)
Sociodemographic characteristics						
Age, median (IQR)	85 (85.1–85.1)	82 (80.5–84.0)	85 (84.8–85.3)	85 (85.2–85.8)	87 (86.3–88.8)	85 (85.1–85.9)
Female, *n* (%)	363 (66.4)	113 (56.8)	175 (50.4)	453 (60.4)	295 (56.2)	1 413 (59.2)
Comorbidities						
ACVD, *n* (%)	252 (46.5)	132 (66.7)	209 (61.1)	414 (55.3)	111 (21.2)	1 126 (47.5)
Diabetes, *n* (%)	89 (16.3)	62 (31.2)	55 (15.9)	110 (15.1)	97 (18.5)	416 (17.6)
Malignancy, *n* (%)	93 (17.1)	31 (15.7)	76 (22.9)	46 (6.1)	100 (19.3)	347 (14.6)
No ACVD, diabetes, malignancy	207 (38.3)	44 (22.2)	93 (27.2)	277 (37.2)	258 (49.8)	879 (37.5)
≥2 of ACVD, diabetes, malignancy	89 (16.4)	61 (30.8)	76 (22.0)	100 (13.2)	41 (7.9)	367 (15.5)
Medication						
Lipid lowering medication, *n* (%)	6 (1.1)	87 (46.3)	161 (47.9)	309 (41.2)	115 (22.0)	684 (29.3)
Laboratory measurements						
Total cholesterol, mean (*SD*), mmol/L	5.7 (1.1)	4.9 (1.1)	5.1 (1.1)	4.8 (1.2)	5.2 (0.8)	5.2 (1.1)
HDL-C, mean (*SD*), mmol/L	1.3 (0.4)	1.5 (0.4)	1.6 (0.4)	1.5 (0.4)	1.5 (0.4)	1.5 (0.4)
LDL-C, mean (*SD*), mmol/L	3.7 (1.0)	2.8 (1.0)	2.9 (0.9)	2.7 (1.0)	3.0 (0.7)	3.0 (1.0)
Triglycerides, median (IQR), mmol/L	1.3 (1.0–2.0)	1.3 (1.0–1.6)	1.2 (1.0–1.6)	1.3 (0.9–1.7)	1.2 (0.8–1.6)	1.3 (0.9–1.7)
Functional parameters						
Disability in ADL, questionnaire	GARS	NEADL	NEADL	ADL sum scores	Lawton iADL	n/a
Disability in ADL, median (IQR)	28 (21–40)	19 (16–21)	19 (17–20)	3 (1–7)	5 (4–5)	n/a
Mini-Mental State Examination, median (IQR)	26 (22–28)	28 (26–29)	28 (26–29)	28 (25–29)	27 (25–29)	27 (25–29)
Grip strength, kg, median (IQR)						
Female	20 (16–22)	19 (17–23)	18 (15–22)	15 (12–19)	16 (14–19)	17 (14–20)
Male	30 (26–36)	31 (27–36)	31 (27–34)	28 (22–33)	25 (22–28)	28 (23–33)

*Notes:* ACVD = atherosclerotic cardiovascular disease; ADL = activities of daily living; GARS = Groningen activity restriction scale; HDL-C = high-density cholesterol; IQR = interquartile range; LDL-C = low-density cholesterol; n/a = not applicable; NEADL = Nottingham extended activities of daily living; *SD* = standard deviation.

### Individual Markers of Fitness

In Supplementary Table 2, the cohort-specific cutoff values of the tertiles of the individual markers of fitness and distribution of participants are presented. The majority of the groups were approximately the intended size (33% of the cohort), except for the TOOTH cohort, in which 67.7% of the participants scored the maximum score on the functional ability questionnaire.

### Composite Fitness Score

The composite fitness score was calculated for 97.1% (*n* = 2 317) of the study population, with 2.9% (*n* = 68) missing. In total, 994 (42.9%) participants had a high composite fitness score, and 694 (30.0%) had a low composite fitness score. The remaining 629 (*n* = 27.1%) participants did not meet the criteria of the high or low composite fitness score. The characteristics of the participants stratified by composite fitness score are shown in Table 2. Compared to participants with a high-composite fitness score, participants with a low-composite fitness score were more likely to have a history of ACVD, diabetes or malignancy, or to have a combination of ≥2 these diseases. They were also more likely to use lipid-lowering medication. The group with a low-composite fitness score had lower LDL-C, HDL-C, and total cholesterol levels and higher triglycerides levels. Participants with a low-composite fitness score were more likely to die during the 5-year follow-up (*n* = 421 deaths [60.7%]), and had shorter survival time (median 1 400 days [IQR 661–1 826]) compared to the participants with a high composite fitness score (*n* = 248 deaths [24.9%] and median survival time 1 826 days [IQR 1 588–1 826]).

**Table 2.** Characteristics of Participants Stratified by Composite Fitness Score^†^ at Baseline

	Level of Composite Fitness Score^†^		
	High (*n* = 994)	Low (*n* = 694)	*p* Value
Sociodemographic characteristics			
Age, median (IQR)	85.3 (85.1–86.1)	85.3 (85.1–85.8)	*.04
Female, *n* (%)	598 (60.2)	414 (59.7)	.83
Morbidity			
ACVD (%)	276 (27.8)	453 (65.3)	*< .01
Diabetes, *n* (%)	92 (9.3)	198 (28.8)	*< .01
Malignancy, *n* (%)	99 (10.0)	140 (20.3)	*< .01
Low (no ACVD, diabetes, malignancy)	598 (59.9)	136 (19.9)	*< .01
High (≥2 of ACVD, diabetes, malignancy)	65 (6.6)	210 (30.5)	*< .01
Medication			
Lipid lowering drugs, *n* (%)	236 (24.0)	232 (33.7)	*< .01
Laboratory measurements			
Cholesterol, mean (*SD*), mmol/L	5.3 (1.1)	5.0 (1.1)	*< .01
HDL-C, mean (*SD*), mmol/L	1.5 (0.4)	1.4 (0.4)	*< .01
LDL-C, mean (*SD*), mmol/L	3.2 (1.0)	2.9 (1.0)	*< .01
Triglycerides median (IQR), mmol/L	1.2 (0.9–1.7)	1.3 (1.0–1.8)	*.02
Functional parameters, median (IQR)			
Functional ability, median (IQR)	cohort specific	cohort specific	—
MMSE, median (IQR)	29 (27–29)	24 (20–27)	*< .01
Grip strength, kg, median (IQR)			
Female	20 (17–22)	13(11–16)	*< .01
Male	32 (26–36)	23 (20–28)	*< .01
Survival			
Median survival time contributed, days, (IQR)	1 826 (1 588–1 826)	1 400 (661–1 826)	*< .01
Number of deaths during follow-up, (%)	248 (24.9)	421 (60.7)	*< .01

*Notes:* ACVD = atherosclerotic cardiovascular disease; HDL-C = high-density cholesterol; IQR = interquartile range; LDL-C = low-density cholesterol; MMSE = Mini-Mental State Examination; *SD* = standard deviation. Results of independent sample *t* tests or Mann-Whitney U-tests for continues variables, and the Chi-square tests for categorical variables.

^†^Composite fitness score: Composite score of the 4 markers (functional ability, cognitive function, grip strength, and morbidity). High composite fitness score: having ≥2 of the 4 markers as high level of fitness. Low composite fitness score: having ≥2 of the 4 markers as low level of fitness. Participants that did not meet these criteria, or with 2 markers of each (high and low level of fitness) were classified as rest group.

*Significance level *p* < .05.

### LDL-C and 5-Year All-Cause Mortality

**Figure 1.**
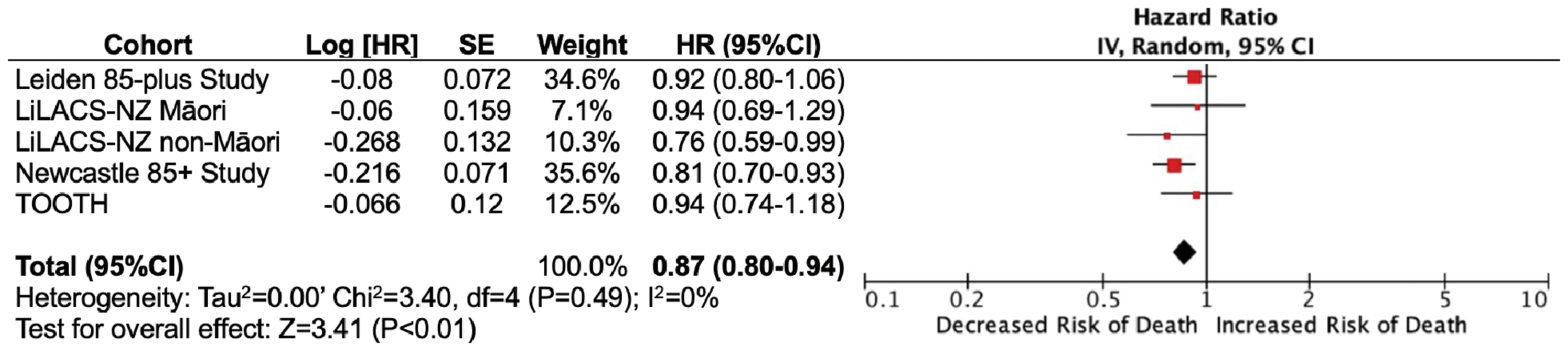
Five-year all-cause mortality risk according to LDL-C level per 1.0 mmol/L in older adults, adjusted for age, gender, and use of lipid-lowering medication. LDL-C = low-density cholesterol.

During the 5-year follow-up, 933 (39.1%) of the participants died. Consistent in all cohorts, there was an inverse association between LDL-C and 5-year mortality risk, pooled HR 0.87 (95% CI: 0.80–0.94). Within the cohorts, the HR ranged from 0.76 (95% CI: 0.59–0.99) in the LiLACS-NZ non-Māori cohort, to HR 0.94 (95% CI: 0.69–1.29) in the LiLACS-NZ Māori cohort, as shown in Figure 1.

#### Individual markers of fitness

**Figure 2.**
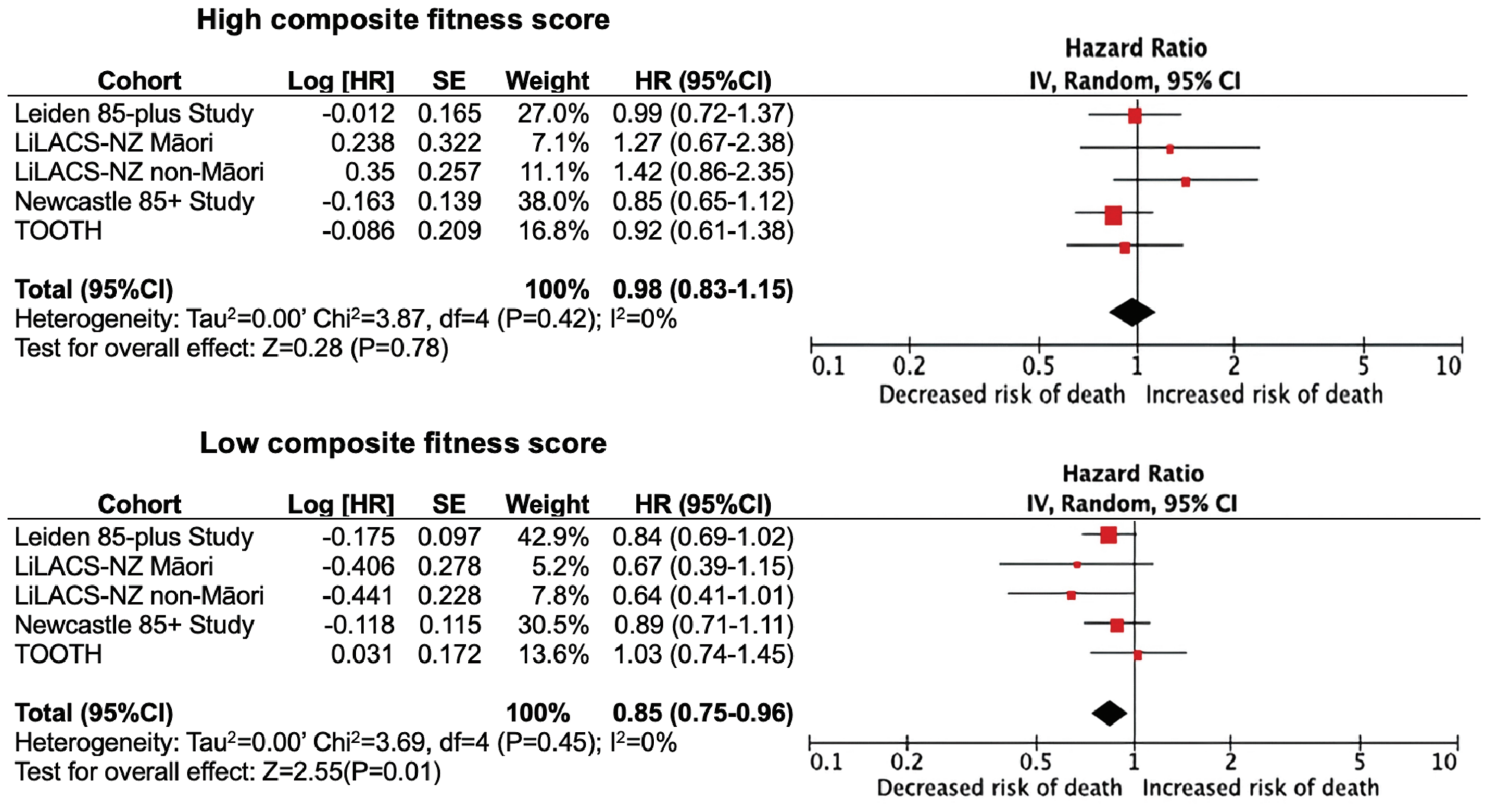
Five-year all-cause mortality risk according to LDL-C level per 1.0 mmol/L, in older adults, adjusted for age, gender, and use of lipid-lowering medication, stratified on composite fitness score. LDL-C = low-density cholesterol.

In Table 3, the 5-year all-cause mortality pooled HRs for 1.0 mmol/L increase in LDL-C, stratified by markers of fitness (high vs low) are presented. There was no association between LDL-C and mortality in the strata with high-functional ability scores (HR 1.00, 95% CI: 0.77–1.29; *p* = .98), while in the groups with low functional ability low LDL-C was associated with a decreased risk of mortality (HR 0.88 [95% CI: 0.78–0.98]; *p* = 0.03, *p* for subgroup difference 0.38). In line with these results, there was no association between LDL-C and mortality in strata with high cognitive function (HR 1.00 [95% CI: 0.80–1.26]; *p* = .98), while in the group with low cognitive function scores low LDL-C was associated with a decreased risk of mortality (HR 0.81 [95% CI: 0.71–0.92]; *p* < .01, *p* for subgroup difference 0.11). Results were mostly consistent across the cohorts (Supplementary Table 3). Moreover, the results of the composite fitness score, in relation to the association between LDL-C and mortality as shown in Figure 2, were also homogeneous between the cohorts. The pooled HR found in the high and low strata of grip strength and morbidity were comparable to the unstratified HR, with no difference between the strata (*p* for subgroup difference for grip strength *p* = .98, and for morbidity *p* = .94).

**Table 3.** Five-Year All-Cause Mortality, Pooled Hazard Ratios for 1.0 mmol/L Increase in LDL-C, Stratified by Markers of Fitness

	High Composite Fitness Score		Low Composite Fitness Score		
Individual Markers of Fitness	Pooled HR (95% CI)	*p* Value	Pooled HR (95% CI)	*p* Value	χ^2^ Test for Subgroup Difference
Functional ability	1.00 (0.77–1.29)^‡^	.98	0.88 (0.78–0.98)	*.03	0.38
Cognitive function	1.00 (0.80–1.26)^‡^	.98	0.81 (0.71–0.92)	*< .01	0.11
Grip strength	0.86 (0.70–1.05)	.14	0.86 (0.72–1.02)	.09	0.98
Morbidity	0.87 (0.74–1.02)	.09	0.88 (0.73–1.06)	.17	0.94
Composite fitness score^†^	0.98 (0.83–1.15)	.78	0.85 (0.75–0.96)	*.01	0.19

*Notes*: Results from Cox proportional-hazards regression models presented as a pooled hazard ratio with 95% confidence intervals (95% CI) for 1 mmol/L increase in LDL-C. Pooling was done by using random-effects models with inverse-variance weighting. Models were corrected for age and gender and lipid lowering treatment. LDL-C = low-density lipoprotein cholesterol.

*Significance level *p* < .05.

^†^Composite fitness score: composite score of the 4 markers of fitness (functional ability, cognitive function, grip strength, and morbidity). High composite fitness score: having ≥2 of the 4 markers as high level of fitness. Low composite fitness score: having ≥2 of the 4 markers as low level of fitness. Participants that did not meet these criteria, or with 2 markers of each (high and low level of fitness) were classified as rest group.

^‡^40%–44%. When not shown or not otherwise labeled: *I*^*2*^ < 40%.

#### Composite fitness score

The forest plot in Figure 2 shows the associations between LDL-C level measured at baseline and 5-year mortality risk, stratified on the composite fitness score (adjusted for age, gender, and lipid-lowering medication). Results were consistent across the 5 cohorts. In the participants with a high composite fitness score, we found no evidence of an association between LDL-C and mortality (HR 0.98 [95% CI: 0.83–1.15]; *p* = .78), while in participants with a low-composite fitness score, a 1 mmol/L increase in LDL-C was associated with a lower mortality risk (HR 0.85 [95% CI: 0.75–0.96]; *p* = .01; *p* for subgroup difference 0.19).

### Sensitivity Analyses

The association between LDL-C and mortality was similar in participants with and without ACVD. Also, the effect of the composite fitness score on the association did not materially change when we additionally stratified on lipid lowering treatment (Supplementary Figures 1 and 2). Findings were similar for total cholesterol.

## Discussion

In this 2-stage IPD meta-analysis, there was an inverse association between baseline LDL-C and all-cause mortality, which was most pronounced in participants with a low-composite fitness score. This trend of effect modification by the composite fitness score was similar for participants with and without lipid-lowering medication.

### Previous Studies

Within the BELFRAIL study (8) it was earlier investigated whether the presence of frailty modifies the association between cardiovascular risk factors and mortality, in a cohort of community-dwelling older adults. No association between LDL-C and mortality was found, nor was a difference in LDL-C between the frail and robust subgroups. There are several explanations for this difference in findings. First, it could be related to the way the subgroups were defined (ie, based on composite fitness score vs frailty). Although, both studies used self-reported and performance measures to distinguish the fittest (or most robust) subjects from those with multiple vulnerabilities. Second, in the current study, participants with intermediate fitness scores were excluded from the subgroup comparisons. This increased the contrast between the subgroup and made it more likely to detect potential differences. It could also explain why the robust and frail subjects in the BELFRAIL study had similar LDL-C levels, while in our study participants with a low composite fitness score had significantly lower LDL-C levels compared to those with a high composite fitness score (Table 2). This latter observation is consistent with previous studies that observed that lower LDL-C levels were associated with more comorbidities, poorer somatic health, cognitive impairment, and higher disability (25). Third, the higher statistical power of this IPD meta-analysis design may also have contributed to the differences in findings.

### Clinical Importance

Results of this study confirm that in very old adults lower LDL-C is associated with higher mortality risk, and the observed trend of effect modification by the composite fitness score suggests that fitness may account for some of the variation in the association that was seen by others (6). These findings raise many intriguing questions, ranging from “How can we causally explain the inverse association,” to “Can we use the composite fitness score to identify certain subgroups who benefit more from LDL-C reduction than others?” However, this is outside of the scope and methodology of this observational study. More research is needed to further explore the influence of fitness (or related concepts) on the predictive value of LDL-C, and how to make the translation to clinical practice. The observation that the trend of effect modification was present in some, but not all of the individual markers of fitness raises the question of which marker of fitness is most important. Ultimately randomized controlled trials about starting and discontinuing cholesterol-lowering medication in older people will give more guidance in clinical practice. The results of this study emphasize the need to include less vital older people in these trials.

### Strengths and Limitations

A key strength of this study is that it combined individual data of 5 substantial cohorts of very old adults from different geographical locations, and diverse cultures, adding to the generalizability of the results. In addition, the 2-stage IPD meta-analysis design allowed us to take into account unmeasured differences between the cohorts (including socio-cultural and health-economic differences) and to combine the data despite methodologic heterogeneity between the studies.

There are some limitations that have to be considered when interpreting the results of this study. First, we performed analyses on observational data, therefore no causal inference can be made. Therefore, our results should not be interpreted as evidence that in some people lowering LDL-C increases mortality risk. One could hypothesize that the found association is the reflection of an epiphenomenon where a possibly genetic pathophysiological abnormality causes an increased mortality risk and decrease in levels of LDL-C in parallel for some older people (26). Alternatively, the found association might be a reflection of reversed causality, and low LDL-C could be a marker of debilitation or of underlying health problems that are responsible for the increased mortality risk (7,27,28). Second, we only included participants who consented to blood sample collection, selection bias might have occurred. The consistency of the finding across the cohorts is however reassuring. Third, fitness was operationalized by four individual markers. There may also be other chronic diseases and additional individual markers of fitness such as nutritional status that could help to further differentiate levels of fitness. Fourth, functional ability was measured with different tools. The concordance between the different tools is unknown. However, by using cohort-specific tertiles, we identified the best and worst functioning within each cohort and therefore, we were able to pool the data. Fifth, across the cohorts, LDL-C was measured by different methods and included both fasting and nonfasting samples. We consider it unlikely that this impacted the results, as we used a 2-stage IPD meta-analysis instead of a 1-stage IPD meta-analysis to pool the HRs to account for this potential source of bias, and in the prediction of all-cause mortality, nonfasting LDL-C has similar prognostic value to that of fasting LDL-C (29).

## Conclusion

In this very old population, there was an inverse association between baseline LDL-C and all-cause mortality, which was most pronounced in participants with a low-composite fitness score. The results of this observational study add a small piece of evidence to unravel the complex relationship between LDL-C and mortality in older adults. More research is needed to investigate important questions on the underlying causal mechanisms and clinical and ethnic-specific implications.

## Supplementary Material

glad148_suppl_Supplementary_File
