# Supplementary material for: The Role of a Composite Fitness Score in the Association Between Low-Density Cholesterol and All-Cause Mortality in Older Adults: An Individual Patient Data Meta-Analysis
Source: J Gerontol A Biol Sci Med Sci. 2023 Jun 14;78(9):1708–16. doi: 10.1093/gerona/glad148 (PMC10460558; doi:10.1093/gerona/glad148)
Supplement: glad148_suppl_Supplementary_File [file glad148_suppl_supplementary_file.pdf]

Supplementary file

eTable 1

eTable 2

eTable3

eFigure 1

eFigure 2

eTable 1. Details on variables used in the analysis

Variable	Description
**Diabetes Mellitus (DM)**	We defined DM (yes/no) in all four studies as a self- and/or general practitioner reported history of DM, the use of antidiabetic medication or an abnormal blood glucose level at baseline.
**History of Atherosclerotic Cardiovascular Diseases (ACVD)**	In the Leiden 85-plus Study, the history of ACVD was available from physician-reported history and electrocardiogram (ECG) data. In the LiLACS NZ study, the CVD data was obtained from a self-reported history, screening of general practitioner (GP) and hospitalization records, and ECGs.(1) In the Newcastle 85+ Study, GP records were screened and ECGs were made. In TOOTH, a history of CVD was obtained from a personal interview and the medical reports brought to that interview.
**Malignancy**	In the Leiden 85-plus Study, the history of malignancy was available from physician-reported history data. In the LiLACS NZ study, the malignancy data was obtained from a self-reported history, screening of general practitioner (GP) and hospitalization records. Non-melanoma skin cancers were excluded. In the Newcastle 85+ Study, GP records were screened, non-melanoma skin cancers [squamous cell, basal cell and skin cancers otherwise specified] and time since cancer > 5 years ago, were excluded. In TOOTH, a history of malignancy was obtained from a personal interview and the medical reports brought to that interview.
**Grip Strength (GS)**	A Jamar hand dynamometer (Sammons Preston INC., Illinois, USA) was used in the Leiden 85-plus Study. A Takei hand dynamometer Grip-D (Takei Scientific Instruments Co., Niigata-City, Japan) was used in the LiLACS NZ and Newcastle 85+ study. In TOOTH, a Tanita 6103 handheld dynamometer (Tanita cooperation, Tokyo, Japan) was utilized. The average value of two and the highest value of three measurements of the dominant hand was used in TOOTH and the Leiden 85-plus Study, respectively. The LiLACS NZ study obtained the average value of three measurements of the strongest hand. In the Newcastle 85+ Study, the mean recording of four measurements alternating between dominant and non-dominant hand were used
**Functional ability**	In the Leiden 85-plus Study, ADL was measured with the Groningen Activity Restriction Scale (GARS).(2) The GARS consists of 18 items scored between 1 (fully independent) and 4 (only with help), adding up to a maximal score of 72 (worst). The LiLACS NZ study utilized the Nottingham Extended Activities of Daily Living (NEADL) questionnaire.(3) The NEADL scores 22 items and ranges from 0 (worst) to 22 (optimal). In the Newcastle 85+ study, ADL was evaluated with a sum score based on 17 activities with total scores ranging from 0 (optimal) to 17 (worst). For TOOTH, the Lawton Instrumental Activities of Daily Living was utilized, with a score ranging from 0 (worst) to 5 (optimal) based on 5 items.(4)

References etable 1

1. Teh R, Doughty R, Connolly M, Broad J, Pillai A, Wilkinson T, et al. Agreement between self-reports and medical records of cardiovascular disease in octogenarians. Journal of clinical epidemiology. 2013;66(10):1135-43. DOI: 10.1016/j.jclinepi.2013.05.001
2. Kempen GI, Miedema I, Ormel J, Molenaar W. The assessment of disability with the Groningen Activity Restriction Scale. Conceptual framework and psychometric properties. Soc Sci Med. 1996;43(11):1601-10. DOI: 10.1016/s0277-9536(96)00057-3
3. Essink-Bot ML, Krabbe PF, Bonsel GJ, Aaronson NK. An empirical comparison of four generic health status measures. The Nottingham Health Profile, the Medical Outcomes Study 36-item Short-Form Health Survey, the COOP/WONCA charts, and the EuroQol instrument. Med Care. 1997;35(5):522-37. DOI: 10.1097/00005650-199705000-00008
4. Lawton MP, Brody EM. Assessment of older people: self-maintaining and instrumental activities of daily living. Gerontologist. 1969;9(3):179-86.

eTable 2 Cut-off values for the tertiles of the individual markers of fitness and distribution of participants

	Leiden-85 plus	LiLACS-NZ		Newcastle 85+	TOOTH	Combined
		a. Māori	b. non-Māori			
**Individual markers of fitness**						
**Functional ability**						
Questionnaire	GARS	NEADL	NEADL	ADL sum	Lawton iADL	
T3 scores indicating high functional ability	≤ 23	≥ 21	≥ 20	≤ 1	5	
Participants in T3, n (%)	192 (35.2)	47 (25.3)	129 (38.4)	267 (35.2)	344 (67.7)	979 (41.9)
T1 scores indicating low functional ability	≥ 35	≤ 17	≤17	≥6	≤ 4	
Participants in T1, n (%)	178 (32.6)	63 (33.9)	103 (30.7)	243 (32.0)	164 (32.3)	751 (32.2)
**Cognitive function (MMSE)**						
T3 scores indicating high cognitive function	≥ 28	≥ 29	≥ 29	≥ 29	≥ 29	
Participants in T3, n (%)	194 (35.6)	55 (29.4)	115 (35.1)	279 (36.4)	186 (36.1)	829 (35.4)
T1 scores indicating low cognitive function	≤ 23	≤ 26	≤ 27	≤ 26	≤ 25	
Participants in T1, n (%)	163 (29.9)	55 (29.4)	133 (40.5)	272 (35.5)	169 (32.8)	792 (33.8)
**Grip strength**						
<u>Males</u>						
T3 scores indicating strongest grip strength	≥ 34.0	≥ 33.5	≥ 33.5	≥ 31.0	≥ 26.6	
Participants in T3, n (% of males)	61 (33.2)	28 (32.6)	59 (34.3)	97 (32.3)	75 (32.6)	319 (32.9)
T1 scores indicating weakest grip strength	≤ 26	≤ 28.10	≤ 27.70	≤ 23.90	≤ 22.40	
Participants in T1, n (% of males)	61 (33.2)	29 (33.7)	57 (33.1)	102 (34.0)	74 (32.2)	319 (32.9)
<u>Females</u>						
T3 scores indicating strongest grip strength	≥ 22.0	≥ 21.9	≥ 20.5	≥ 17.30	≥ 18.30	
Participants in T3, n (% of females)	103 (28.4)	38 (33.6)	56 (32.0)	152 (32.5)	93 (31.5)	438 (31.3)
T1 scores indicating weakest grip strength	≤ 16	≤ 17.20	≤ 16.20	≤ 13.40	≤ 14.50	
Participants in T1, n (% of females)	134 (36.9)	37 (32.7)	60 (34.3)	162 (34.7)	98 (33.2)	483 (34.5)
**Morbidity**						
Low (no ACVD, diabetes, malignancy)	207 (38.3)	44 (22.2)	93 (27.2)	277 (37.2)	258 (49.8)	879 (37.5)
High (≥2 of ACVD, diabetes, malignancy)	89 (16.4)	61 (30.8)	76 (22.0)	100 (13.2)	41 (7.9)	367 (15.5)

ACVD: atherosclerotic cardiovascular disease, GARS: Groningen Activity Restriction Scale. HDL-C: High-density cholesterol. IQR: interquartile range. LDL-C low-density cholesterol. NEADL: Nottingham Extended Activities of Daily Living.

62

eTable 3. All-cause 5 years mortality, un-pooled hazard ratios per 1.0 mmol/l increase in LDL-C, stratified by individual markers of fitness

		Leiden-85 plus		LiLACS-NZ Māori		LiLACS-NZ non- Māori		Newcastle 85+		TOOTH	
	**Level of fitness**	**HR (95%CI)**	***P* value**	**HR (95%CI)**	***P* value**	**HR (95%CI)**	***P* value**	**HR (95%CI)**	***P* value**	**HR (95%CI)**	***P* value**
**Total study population**		0.92 (0.80-1.06)	0.23	0.94 (0.69-1.29)	0.71	0.77 (0.59-0.99)	*0.04	0.81 (0.70-0.93)	*<0.01	0.94 (0.74-1.18)	0.58
**Functional ability**	high[a]	1.15 (0.82-1.60)	0.41	0.69 (0.16-2.97)	0.62	1.67 (0.95-2.96)	0.08	0.77 (0.57-1.04)	0.09	0.89 (0.63-1.27)	0.52
	low	0.84 (0.70-1.00)	*0.05	0.71 (0.42-1.19)	0.19	0.71 (0.47-1.07)	0.10	0.96 (0.77-1.12)	0.72	1.00 (0.74-1.36)	0.99
**Cognitive function**	high[a]	0.76 (0.55-1.05)	0.10	1.78 (0.86-3.67)	0.12	0.92 (0.56-1.49)	0.73	0.98 (0.77-1.25)	0.88	1.29 (0.84-1.96)	0.25
	low	0.84 (0.68-1.03)	0.09	0.99 (0.54-1.81)	0.96	0.75 (0.51-1.12)	0.16	0.73 (0.58-0.92)	*<.0,01	0.93 (0.65-1.34)	0.70
**Grip strenght**	high	0.92 (0.66-1.28)	0.64	1.08 (0.63-1.86)	0.78	1.18 (0.71-1.95)	0.53	0.73 (0.54-0.97)	*0.03	0.61 (0.35-1.06)	0.08
	low	0.91 (0.75-1.11)	0.36	0.94 (0.54-1.62)	0.82	0.47 (0.29-0.78)	*0.00	0.87 (0.71-1.06)	0.17	1.00 (0.73-1.38)	0.99
**Morbidity**	high[b]	0.81 (0.59-1.11)	0.19	1.05 (0.47-2.36)	0.91	1.13 (0.64-2.02)	0.67	0.83 (0.65-1.06)	0.14	0.92 (0.62-1.35)	0.66
	low[c]	0.96 (0.72-1.28)	0.77	0.95 (0.50-1.81)	0.87	0.79 (0.48-1.30)	0.35	0.78 (0.54-1.13)	0.19	0.88 (0.48-1.59)	0.67

Note: HR: Hazard ratio

Results from Cox proportional-hazards regression models presented as a pooled hazard ratio with 95% confidence intervals for 1mmol/L increase in LDL-C. Pooling was done by using random-effects models with inverse-variance weighting. Models were corrected for age and gender and lipid lowering treatment.

[a] I^2 = 40-44%. When not shown or not otherwise labeled: I^2 <40%.
[b] high level of fitness on the morbidity variable indicates low morbidity= no atherosclerotic cardiovascular disease, diabetes or malignancy
[c] low level of fitness on the morbidity variable indicates high morbidity ≥2 of atherosclerotic cardiovascular disease, diabetes, malignancy

63

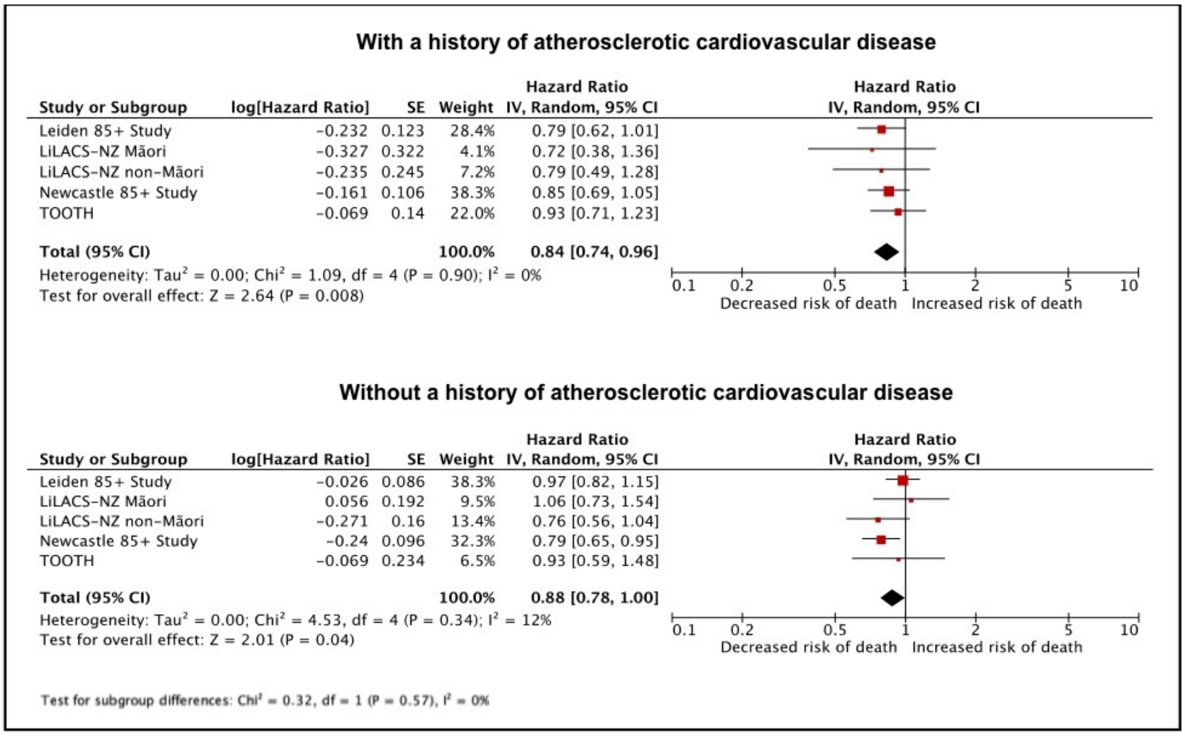

eFigure 1. All-cause 5-year mortality risk according to LDL-C mmol/L, adjusted for age, gender, and lipid-lowering medication use, stratified by history of atherosclerotic cardiovascular disease

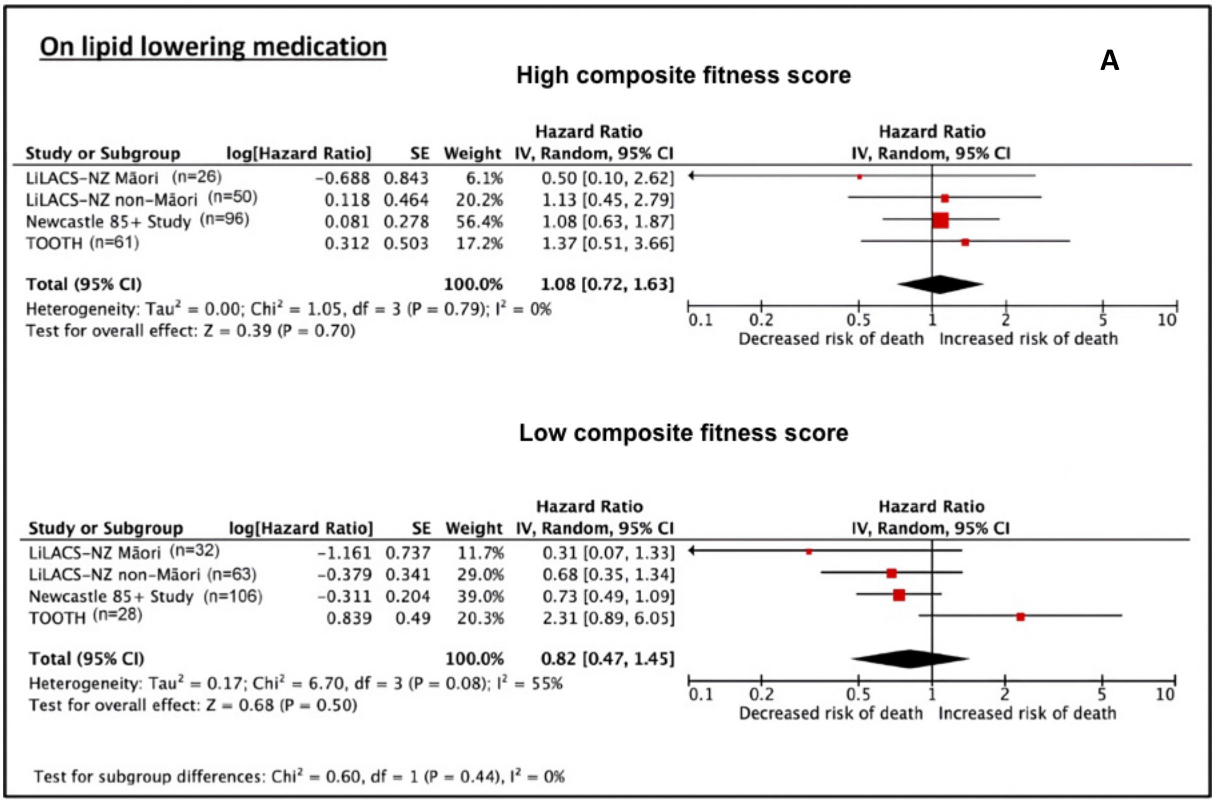
On lipid lowering medication
A
High composite fitness score
Study or Subgroup | log[Hazard Ratio] | SE | Weight | Hazard Ratio IV, Random, 95% CI
LiLACS-NZ Māori (n=26) -0.688 0.843 6.1% 0.50 [0.10, 2.62]
LiLACS-NZ non-Māori (n=50) 0.118 0.464 20.2% 1.13 [0.45, 2.79]
Newcastle 85+ Study (n=96) 0.081 0.278 56.4% 1.08 [0.63, 1.87]
TOOTH (n=61) 0.312 0.503 17.2% 1.37 [0.51, 3.66]
Total (95% CI) 100.0% 1.08 [0.72, 1.63]
Heterogeneity: Tau² = 0.00; Chi² = 1.05, df = 3 (P = 0.79); I² = 0%
Test for overall effect: Z = 0.39 (P = 0.70)
Hazard Ratio IV, Random, 95% CI
0.1 0.2 0.5 1 2 5 10
Decreased risk of death Increased risk of death
Low composite fitness score
Study or Subgroup | log[Hazard Ratio] | SE | Weight | Hazard Ratio IV, Random, 95% CI
LiLACS-NZ Māori (n=32) -1.161 0.737 11.7% 0.31 [0.07, 1.33]
LiLACS-NZ non-Māori (n=63) -0.379 0.341 29.0% 0.68 [0.35, 1.34]
Newcastle 85+ Study (n=106) -0.311 0.204 39.0% 0.73 [0.49, 1.09]
TOOTH (n=28) 0.839 0.49 20.3% 2.31 [0.89, 6.05]
Total (95% CI) 100.0% 0.82 [0.47, 1.45]
Heterogeneity: Tau² = 0.17; Chi² = 6.70, df = 3 (P = 0.08); I² = 55%
Test for overall effect: Z = 0.68 (P = 0.50)
Hazard Ratio IV, Random, 95% CI
0.1 0.2 0.5 1 2 5 10
Decreased risk of death Increased risk of death
Test for subgroup differences: Chi² = 0.60, df = 1 (P = 0.44), I² = 0%

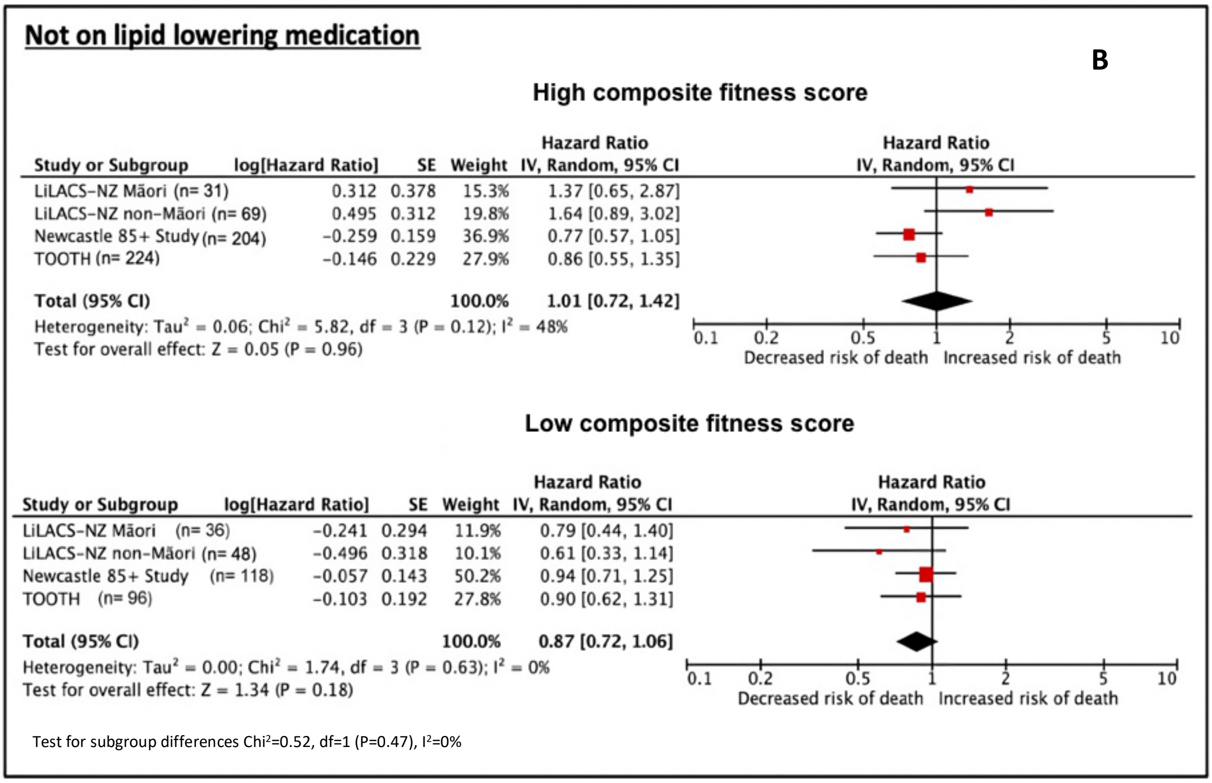
Not on lipid lowering medication
B
High composite fitness score
Study or Subgroup | log[Hazard Ratio] | SE | Weight | Hazard Ratio IV, Random, 95% CI
LiLACS-NZ Māori (n= 31) 0.312 0.378 15.3% 1.37 [0.65, 2.87]
LiLACS-NZ non-Māori (n= 69) 0.495 0.312 19.8% 1.64 [0.89, 3.02]
Newcastle 85+ Study (n= 204) -0.259 0.159 36.9% 0.77 [0.57, 1.05]
TOOTH (n= 224) -0.146 0.229 27.9% 0.86 [0.55, 1.35]
Total (95% CI) 100.0% 1.01 [0.72, 1.42]
Heterogeneity: Tau² = 0.06; Chi² = 5.82, df = 3 (P = 0.12); I² = 48%
Test for overall effect: Z = 0.05 (P = 0.96)
Hazard Ratio IV, Random, 95% CI
0.1 0.2 0.5 1 2 5 10
Decreased risk of death Increased risk of death
Low composite fitness score
Study or Subgroup | log[Hazard Ratio] | SE | Weight | Hazard Ratio IV, Random, 95% CI
LiLACS-NZ Māori (n= 36) -0.241 0.294 11.9% 0.79 [0.44, 1.40]
LiLACS-NZ non-Māori (n= 48) -0.496 0.318 10.1% 0.61 [0.33, 1.14]
Newcastle 85+ Study (n= 118) -0.057 0.143 50.2% 0.94 [0.71, 1.25]
TOOTH (n= 96) -0.103 0.192 27.8% 0.90 [0.62, 1.31]
Total (95% CI) 100.0% 0.87 [0.72, 1.06]
Heterogeneity: Tau² = 0.00; Chi² = 1.74, df = 3 (P = 0.63); I² = 0%
Test for overall effect: Z = 1.34 (P = 0.18)
Hazard Ratio IV, Random, 95% CI
0.1 0.2 0.5 1 2 5 10
Decreased risk of death Increased risk of death
Test for subgroup differences Chi²=0.52, df=1 (P=0.47), I²=0%

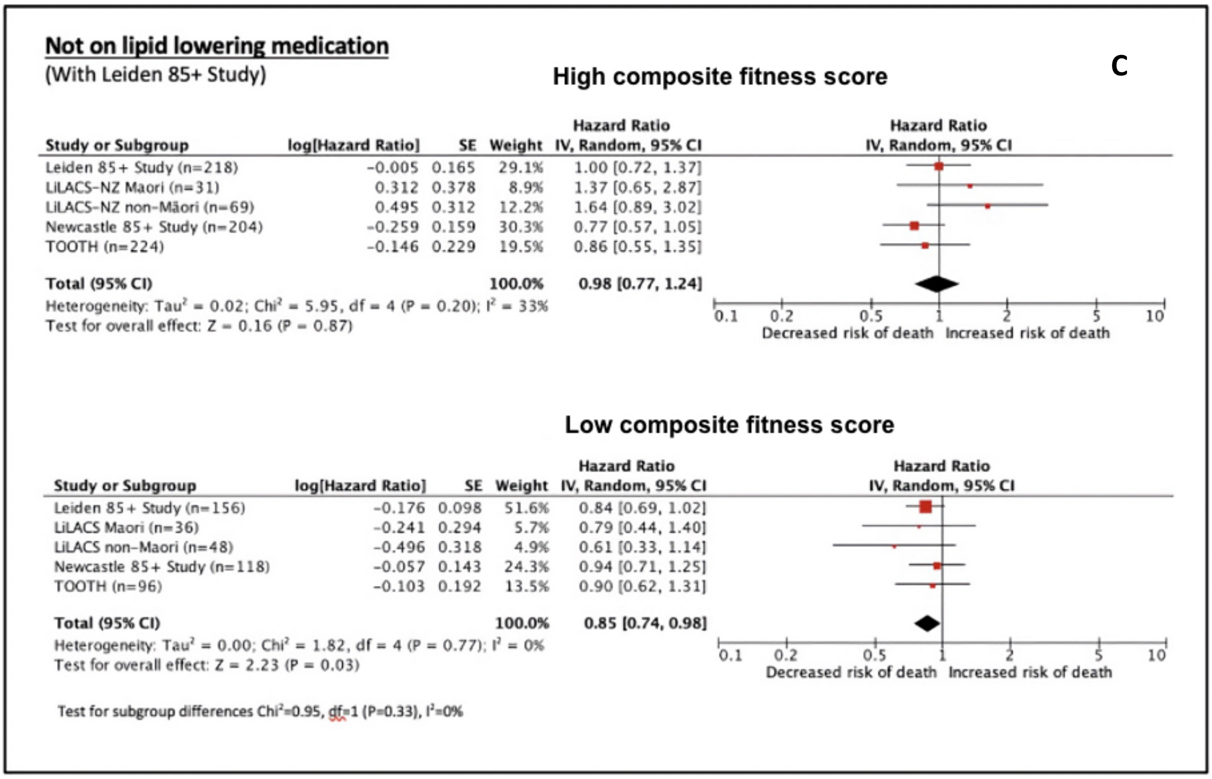

eFigure 2. All-cause 5-year mortality risk according to LDL-C mmol/L, adjusted for age and gender, stratified by composite fitness score, and lipid lowering treatment.

Panel A= on lipid lowering treatment (the Leiden cohort was excluded from this analysis because of too few statin users [n=6]); **Panel B**= participants not on lipid lowering medication, excluding the Leiden 85+ cohort; **panel C**= participants not on lipid lowering medication, including the Leiden 85+ cohort.
